# Inequality in income change among cancer survivors five years after diagnosis: Evidence from a French national survey

**DOI:** 10.1371/journal.pone.0222832

**Published:** 2019-10-03

**Authors:** Caroline Alleaume, Marc-Karim Bendiane, Patrick Peretti-Watel, Anne-Déborah Bouhnik

**Affiliations:** 1 Aix Marseille Univ, INSERM, IRD, SESSTIM, Sciences Economiques & Sociales de la Santé & Traitement de l’Information Médicale, Marseille, France; 2 Aix Marseille Univ, IRD, AP-HM, SSA, VITROME, IHU-Méditerranée Infection, Marseille, France; 3 ORS PACA, Observatoire régional de la santé Provence-Alpes-Côte d’Azur, Marseille, France; Catholic University of Korea College of Medicine, REPUBLIC OF KOREA

## Abstract

Worldwide, around 18 million people receive a cancer diagnosis each year, most of whom survive long enough to face additional cancer-related costs. In France, most costs directly related to cancer are covered by the National Health Insurance Fund, and cancer patients can receive treatments without paying advance fees. In this context, the costs faced by cancer survivors are mostly social costs. Drawing on fundamental cause theory, this study aimed to explore the socially-differentiated evolution of cancer survivor’s income five years after diagnosis. Our study draws on the findings of VICAN5, a French national survey that was conducted in 2015/2016 in a representative sample of 4,174 cancer survivors to obtain information on living conditions five years after diagnosis, and that was restricted to 12 tumour sites accounting for 88% of global cancer incidence in France. We used the multiple imputation method and the Heckman selection model to identify the factors associated with a decrease in household income per consumption unit (HICU), while accounting for missing data. Among survivors still working five years after diagnosis, 17.6% reported lower income at survey than at diagnosis. After adjustment for socio-demographic and medical characteristics, the decrease in HICU was more frequent in women, singles, low educated survivors, and survivors with reduced working time. Finally, subjective measures of income variation and economic well-being were a useful complement to objective measures since 31.6% of cancer survivors still working five years after diagnosis reported a perceived decrease in household income. In conclusion, inequalities in economic well-being persist long after diagnosis in France, and this despite the fact that most cancer-related costs are covered by the French National Health Insurance Fund. Consequently, more attention should be paid to cancer patients with low socio-economic status to help reduce inequalities in post-diagnosis living conditions.

## Introduction

In several countries, studies have found that cancer survivors are more likely than the general population to report financial difficulties [1–3]. Economic decline after a cancer diagnosis has been estimated to be around 7% in Norway [2] and as high as 40% in the United States [4]; likewise, in Canada, an income reduction of 37% has been reported in employed breast cancer survivors [5]. More recently, a Canadian study [6] compared a group of cancer survivors with a control group composed of similarly employed individuals who were never diagnosed with cancer: the income of survivors was found to have decreased by 10%, and this income reduction was shown to be socially differentiated. More generally, a strong association has been documented between economic decline and quality of life degradation in cancer survivors [7–9]. Nevertheless, the economic situation of survivors has also been found to recover over time, namely three years after diagnosis [4,10].

In line with fundamental cause theory [11,12], which has documented a consistent association between socio-economic factors and disparities in health and mortality, studies have shown that low socio-economic status is negatively related to survival and to post-diagnosis quality of life in cancer survivors. Roth et al. have reported that low educated individuals are more likely to develop passive and maladaptive coping strategies in the face of chronic pain [13]. Barbareschi et al. have similarly found that up to one year after diagnosis, high educated individuals have better physical adaptation and role functioning than low educated ones [14]. Other studies have shown that low educational level is the factor most frequently associated with reduced earnings after a cancer diagnosis [2,6,15,16]. In light of these findings, we can presume that social vulnerability, defined as “the susceptibility of a community to the impact of hazards” [17], has a negative impact on cancer survivors, and in particular on their economic situation.

In France, the scientific literature concerning the impact of cancer on survivors’ earnings is limited. This is a serious lacuna, especially since more than three million people over 15 years of age living in the country have received a cancer diagnosis in their lifetime [17], and since working-age individuals account for nearly half of diagnosed cancers [18]. The French National Health Insurance Fund covers almost all costs related to cancer (including transportation costs) on the grounds that it is a long-duration disease; moreover, cancer patients can receive treatments and medications without paying advance fees. Despite such comprehensive coverage, survivors face important social costs in the form of reduced earnings [19,20]. It was corroborated by VICAN2 [21], a French national survey conducted in 2012 among patients to obtain information on living conditions two years after cancer diagnosis. This survey found that more than one in five survivors experienced a decrease in income two years after diagnosis. This decrease, which ranged between 250 and 1,000 euros per month and was mostly due to job loss or working time reduction, was more prevalent in patients who were most affected by the disease and treatment.

In light of the above, we set out to analyse income change among a large sample of cancer survivors in France five years after diagnosis. Drawing on fundamental cause theory, we posited that cancer-related costs are borne differently depending on the socio-economic characteristics of survivors. Our research question was as follows: in the French context, where (almost) all cancer-related costs (including, if applicable, invalidity pension) are covered by the National Health Insurance Fund regardless of one’s situation, can inequalities in social conditions explain differences in income change among cancer-affected households? More specifically, our aim was to document the differential impact of cancer on the economic lives of survivors in a country where the costs faced by cancer patients are mostly social costs.

## Materials and methods

### The VICAN5 survey

#### Targeted population

The VICAN5 survey (derived from “VIe après le CANcer,” French for “Life after cancer”) was carried out among individuals aged between 18 and 82 and living in metropolitan France in 2010, at which time they were diagnosed with a first malignant cancer located in one of 12 common tumour sites that accounting for 88% of global cancer incidence in France [22]. Eligibility was restricted to French-speaking survivors registered with one of the three main French health insurance schemes (Caisse Nationale de l’Assurance Maladie des Travailleurs Salariés (CNAMTS) for salaried workers, Régime Social des Indépendants (RSI) for self-employed workers, and Mutuelle Sociale Agricole (MSA) for farmers), which together cover more than 90% of the French population.

#### Ethics approval and consent to participate

The survey methodology was approved by three national ethics commissions: the CCTIRS (Comité Consultatif sur le Traitement de l’Information en Matière de Recherche dans le Domaine de la Santé, study registered under no 11–143), the ISP (Institute of Public Health, study registered under no C11-63) and the CNIL (French Commission on Individual Data Protection and Public Liberties, study registered under no 911290). Confidentiality is assured for all participants with regard to any personal responses and information provided, as all data collected are anonymized.

#### Data collection

Data used were collected in 2015/2016 from three different sources: 1) a patient questionnaire administered by phone; and 2) the medico-administrative databases of the French National Health Insurance Fund known as the *Système National d’Information Interrégimes de l’Assurance Maladie* (SNIIRAM). Several data on living conditions were collected: socio-demographic characteristics, some of which were gathered at diagnosis (gender, age, educational level, and marital status) and others at survey (place of residence, number and age of dependent children, marital status, number of people living in the household, etc.); treatments received; duration of sick leave; perceived sequelae; professional situation at diagnosis (type of contract, working time, socio-professional category, sector of activity, etc.); professional changes between diagnosis and survey (change of job and/or employer, working time reduction); working conditions at survey; and income-related variables (household income and professional income at diagnosis and at survey, and perceived variation in household income between diagnosis and survey).

Two samples were constituted for data collection. The first, called “primary sample,” was composed of participants who filled the patient questionnaire twice: two years after diagnosis (in 2012) and five years after diagnosis (in 2015). The second sample, called “additional sample” and accounting for approximately 50% of the total sample, was formed by participants who responded only once to the questionnaire, namely five years after diagnosis (in 2015/2016) [22].

#### Weighting procedure

Different weights were applied to the characteristics of survivors (age, national insurance scheme, and tumour site) to ensure representativeness of the target population (see above). To account for potential selection bias, these weights were then adjusted for the characteristics of eligible non-respondents, namely gender, age, national insurance scheme, tumour site, estimated severity of the disease, and social disadvantage index based on place of residence [22].

#### Reported income: Methodological difficulties and related indicators

We encountered two methodological difficulties with respect to income data:

- The first difficulty stemmed from the fact that some individuals reported their income as a range of values. Specifically, participants were asked to provide their accurate income information or, failing that, to select a range of income from the following: 0€ / less than 500€ / between 500€ and less than 1,000€ / between 1,000€ and less than 1,250€ / between 1,250€ and less than 1,500€ / between 1,500€ and less than 2,000€ / between 2,000€ and less than 2,500€ / between 2,500€ and less than 3,000€ / between 3,000€ and less than 5,000€ / between 5,000€ and less than 8,000€ / 8,000€ or more.
- The second difficulty was related to missing data: in some cases, no response was given to the income question.

To exploit the answers given as ranges, we used a method combining bootstrap estimates and multiple imputations [23–25]. Specifically, for each range of income, we estimated the mean income by performing linear regressions on one hundred subsamples taken from the group of survivors who had provided their accurate income information. We then imputed the estimated income for each participant who had given the considered range as an answer. Finally, to ensure estimate consistency, we conducted a sensitivity analysis comparing the distribution of income among survivors who had provided their accurate income information to the global distribution of income (declared + imputed).

When faced with missing data, we performed no imputation, as these data were unlikely to be missing at random. We nonetheless accounted for potential bias in the linear regressions by using the Heckman selection model [26], as explained in the Statistical Analyses section below.

On the basis of reported and estimated incomes, we created the following indicators:

Household income per consumption unit (HICU). This indicator was derived from individual declarations and obtained by dividing household resources (including social benefits) by total household weight. The latter measure, which is commonly used to compare the income of households of different sizes and compositions, was calculated based on the OECD-modified scale [27]. Specifically, we converted the number of individuals per household into a number of consumption units (CU), to which we assigned the following weights: 1 CU to the first adult household member; 0.5 CU to each additional member aged 14 and over; and 0.3 CU to each child under 14.

Variation in HICU. Variation in HICU was obtained by measuring the difference between HICU at diagnosis and HICU at survey. Because this difference has a different impact depending on income at baseline, we calculated the proportion of HICU lost as follows: (HICU at diagnosis–HICU at survey) / HICU at diagnosis. Decrease (or increase) in HICU was defined as a gap between HICU at diagnosis and HICU at survey greater than 10%. The threshold of 10% was selected in order to account for natural variation in income over the five-year period (for instance, due to inflation estimated around 2% per year [28]) and for any margin of error (for instance, due to memory bias).

Perceived variation in household income. Two variables were used to measure survivors’ perception of their economic situation. The first measured perceived change in household income: “Since you were diagnosed with cancer, has your household income?” “Increased a lot,” “increased a little,” “remained the same,” “decreased a little,” or “decreased a lot.” This variable was aggregated into three categories: “increase,” “no change,” and “decrease.” The second variable measured the perceived impact of cancer on professional income: “In your opinion, has the disease caused your professional income to decrease?” “Yes, a lot,” “yes, somewhat,” “yes, a little bit,” “no, not at all.” This variable was aggregated into two categories: “yes” and “no.”

#### Other indicators

Working time reduction. French employment law states that the length of the working week is 35 hours, and that the maximum length of the working day is 10 hours (article L. 212–1 of the Labour Code). In view of this, we defined working time reduction as a reduction of the working week by at least half a day (or four hours) between diagnosis and survey.

Medical characteristics. Some of the tumour sites were gender specific. Indeed, breast, cervical, and uterine cancer concern only women, whereas prostate cancer develops only in men. Accordingly, we constructed a combined variable of gender and gender-specific or non-gender-specific tumour site with the following three modalities: 1) female and female-specific cancer; 2) female and non-gender-specific cancer; and 3) male and male-specific and non-gender-specific cancer. Given the low proportion of men with prostate cancer in our study population, we did not separate the male-specific tumour site (i.e., prostate) from other tumour sites, and consequently did not include the modality “male and male-specific cancer only.”

We subsequently used data from SNIIRAM databases to identify patients who experienced an adverse cancer event after diagnosis and those who had comorbidities. Adverse cancer event was defined as having had metastases, recurrence, or a secondary cancer between diagnosis and survey. Patients who underwent palliative care, chemotherapy, radiotherapy, or targeted therapy at least two years after diagnosis were also considered to have experienced an adverse cancer event. Lastly, comorbidity at diagnosis was measured using a score of individual chronic conditions (excluding cancer) based on data from the SNIIRAM databases [29].

#### Study population

The study population was restricted to cancer survivors who were still working five years after diagnosis. This subsample was used to explore HICU variation in patients who experienced an unexpected decrease in HICU. Participants with missing data on income were included to account for potential selection bias (N = 1,636).

### Statistical analyses

We began by performing Chi-square tests to compare the characteristics of the following groups: survivors who experienced a decrease, no change or an increase in HICU. We also investigated the factors associated with a decrease in HICU, namely socio-demographic characteristics, professional situation, and medical characteristics. Starting from the premise that working time reduction is endogenous to the decrease in HICU, we used a recursive bivariate probit model that accounted for the factors associated with working time reduction and for those associated with a decrease in HICU. Despite the ability of this model to explain working time reduction, it did not seem relevant to our analysis (rho > 0.05). In view of this, we chose to present in this paper the simple probit model, which included working time reduction as an independent variable.

In order to test the potential bias due to missing data, we estimated it in a simultaneous probit equations model identifying factors associated to the decrease in HICU taking into account the selection bias correction [23]. The variables introduced in the selection equation were: socio-demographic characteristics (gender, age, marital status, dependent children (or not), educational level), professional situation (salaried or self-employed status and socio-professional category), perception of economic well-being, and medical characteristics (adverse cancer event, comorbidities). The variable “sample to which respondents belong” (primary or additional sample) was also included to account for memory bias due to non-response. Our assumption was that survivors who answered the questionnaire two years after diagnosis were more likely to respond to the income question at diagnosis than those who answered it five years later.

## Results

**Fig 1:**
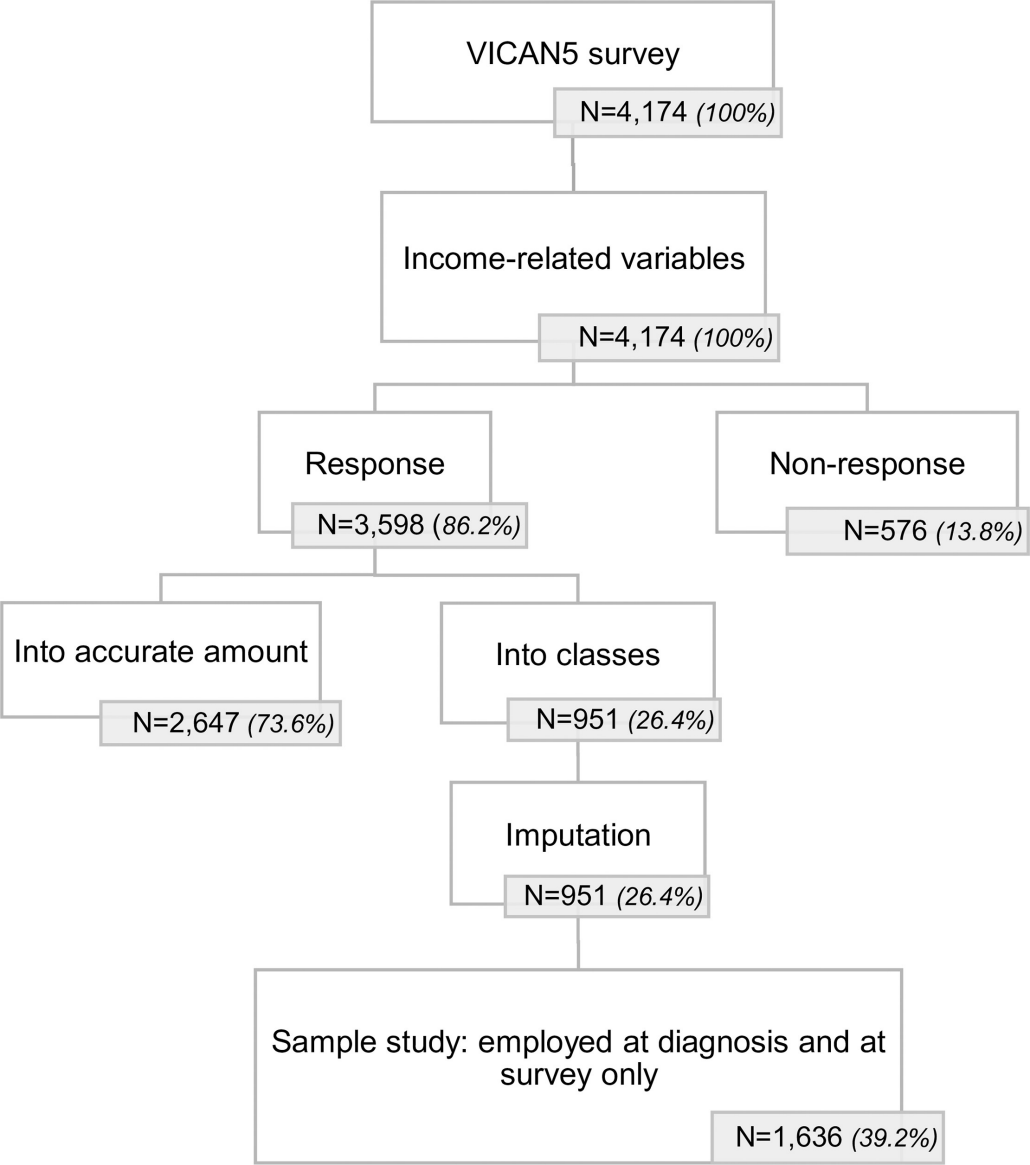
Flow chart of the study population.

As shown in Fig 1, 13.8% of the 4,174 cancer survivors in our study declined to respond to the income question, whether regarding the situation at diagnosis (11.1%) or the situation at survey (9.6%). These data were not missing at random, as they were more prevalent in men (16.9% *versus* 11.9% in women), in survivors aged over 50 (17.5% *versus* 8.8% in survivors aged 40–49 and 6.1% in survivors aged under 40), in singles (23.2% *versus* 11.6% in survivors in a relationship), and in survivors who were working at diagnosis (19.2% *versus* 9.2% in survivors who were not working at diagnosis). These associations were considered for further analysis, as described below.

Among the remaining 3,598 participants who responded to the income question, 26.4% provided a range of values. Their actual income was estimated with the multiple imputation method. The sensitivity analysis showed no statistically significant difference between the distribution of imputed income and the distribution of income declared for each range.

Overall, of all cancer survivors who responded to the income question, 20.5% of cancer survivors reported a decrease in income at survey. Conversely, 46.6% reported an increase, and 32.7% reported no change. As expected, very few survivors who were retired at diagnosis reported a decrease in income five years later. For others, the decrease in income was strongly associated with job loss. While this seemed to confirm our initial assumption, survivors who were still working five years after diagnosis also reported a decrease in income. In view of this, we restricted our subsequent analyses to survivors who were still working five years after diagnosis.

### Variation in HICU among survivors still working five years after diagnosis

Among the study population, more than one in six survivors (17.6%) reported a decrease in income of 10%, whereas 46.7% reported an increase of 10% and 27.7% reported no change. The last 8.0% provided no information on income (Table 1). The average change in HICU were of +220€ per month. This amount was of 773€ less per month for cancer survivors who had a decrease and of 714€ more per month for those who had an increase.

**Table 1:** Variation in HICU between diagnosis and survey (N = 1,636).

	All	Decrease	No change	Increase	Missing
	100%	17.6%	27.7%	46.7%	8.0%
**Amount of variation in HICU **					
Mean (median) HICU in Euros	+220 (+160)	-773 (-500)	+17 (0)	+714 (+533)	-
Median variation in HICU in %	+11.1%	-28.6%	0.0%	+42.9%	-

### Characteristics of survivors who had a decrease in HICU

The study population was mostly composed of women diagnosed with breast cancer (Table 2). The other main characteristics of survivors still working at survey were as follows: age between 40 and 49, being in a relationship, having no dependent children, high educational level, working in the private sector at diagnosis, and holding a permanent contract at diagnosis. At survey, nearly one in four survivors reported a change of job and/or employer after diagnosis, and three in ten reported a reduction in working time.

**Table 2:** Characteristics associated with variation in HICU between diagnosis and survey (N = 1,636).

		All	Variation in HICU Column %			
			Decrease	No change	Increase	Missing
**Socio-demographic characteristics**						
**Gender**			* ****	* ref*.	* ns*	*ns*
	Male	21.0	12.5	22.9	21.5	29.7
	Female	79.0	87.5	77.1	78.5	70.3
**Age at diagnosis**			* ns*	* ref*.	* ns*	* ****
	< 40	20.3	24.6	21.0	19.6	12.0
	> = 40 and < 50	54.3	49.2	55.8	56.3	49.2
	> = 50	25.4	26.2	23.2	24.1	38.8
**Marital status at diagnosis**			* ns*	* ref*.* *	* ns*	* ***
	In a relationship	85.3	90.2	87.0	83.7	77.6
	Single	14.7	9.8	13.0	16.3	22.4
**Dependent children at diagnosis**			* ns*	* ref*.	* ***	*ns*
	Yes	30.4	33.6	34.1	26.9	31.6
	No	69.6	66.4	65.9	73.1	68.4
**Educational level at diagnosis**			* **	* ref*.* *	* **	* ****
	Primary	3.1	4.1	1.6	3.1	6.7
	Secondary	31.0	31.0	27.4	32.3	35.4
	Tertiary	65.9	64.9	71.0	64.6	57.9
**Place of residence**			*ns*	* ref*.* *	*ns*	*ns *
	Rural	25.7	25.5	23.1	27.8	23.6
	Small town (< 200,000 residents)	39.2	38.4	40.1	39.7	34.8
	Big city (> = 200,000 residents)	35.1	36.1	36.8	32.5	41.5
**HICU**			* ns*	* ref*.* *	* ****	* ****
	High (> 3^rd^ quartile)	22.0	35.9	31.6	14.6	1.6
	Intermediate (q1-q3)	48.1	56.7	57.1	45.8	11.7
	Low (< 1^st^ quartile)	23.4	7.4	11.3	39.6	5.0
	Missing	6.5	0	0	0	81.7
**Job characteristics at diagnosis**						
**Sector of activity**			* ns*	* ref*.* *	* ns*	* ***
	Public	23.1	24.4	23.2	24.4	12.3
	Private	76.9	75.6	76.8	75.6	87.7
**Socio-professional category**			* *#	* ref*.* *	* ns*	*ns*
** **	Execution	52.5	58.3	51.3	51.2	51.4
	Supervisors	47.5	41.7	48.7	48.8	48.6
**Type of contract**			* **	* ref*.* *	* ns*	**** *
	Permanent	74.1	70.2	77.6	77.2	52.5
	Fixed term	9.2	12.8	6.9	9.5	7.7
	Self-employed	15.2	15.7	15.2	12.2	31.9
	Missing	1.5	1.3	0.4	1.1	7.9
**Job-related change between diagnosis and survey**						
**Change of job and/or employer after diagnosis**			**** *	* ref*.* *	* **	*ns*
	Yes	24.2	39.1	19.2	24.9	24.0
	No	75.8	69.9	80.8	75.2	76.0
**Working time reduction after diagnosis**			* ****	* ref*.* *	* ns*	* ***
	Yes	30.1	47.0	29.2	26.6	17.2
	No	69.9	53.0	70.8	73.4	82.8
**Medical characteristics**						
**Duration of first sick leave**			*ns *	* ref*.* *	* *#	*** *
** **	None (or < 1 month)	18.0	18.1	18.0	16.2	28.6
	< 6 months	40.7	31.8	38.9	44.3	45.5
** **	between 6 months and 1 year	16.8	17.6	16.2	18.4	7.7
	between 1 and 2 years	16.7	23.6	18.6	14.5	8.3
	> = 2 years	6.3	7.5	7.3	5.0	7.7
	Missing	1.5	1.4	1.1	1.6	2.2
**Tumor site**			* ns*	* ref*.* *	* ns*	* ****
	Breast	55.4	64.4	53.9	54.4	46.8
	Lung and aerodigestive tract	6.0	4.6	4.7	4.3	23.2
	Colon-rectum	6.7	6.8	7.3	6.6	5.6
	Bladder, kidney and prostate	7.4	5.0	8.8	7.1	9.0
	Thyroid	9.8	7.7	8.2	12.5	3.7
	Non-Hodgkin lymphoma	4.4	3.6	5.6	4.3	2.3
	Melanoma	7.2	5.0	7.6	8.0	5.9
	Uterus and cervix	3.1	2.9	3.9	2.8	3.5
**Adverse cancer event between diagnosis and survey**			#	*ref*.* *	**	* ns*
	Yes	17.7	16.0	20.9	14.9	26.4
	No	82.3	84.0	79.1	85.1	73.6

***p value < 0.01%

**p value < 1%

*p value < 5%

^#^p value < 10% (Chi squared test). Each test was performed on a selected group comparing separately paired groups: decrease vs not change (ref.), increase vs not change and missing vs not change.

Note to the reader: 79.0% of survivors who were still working at survey were women, and these represent 87.5% of people who experienced a decrease in HICU.

Comparing respectively the group with decreased HICU and the group with increased HICU to the reference group (without any change in HICU), we found that cancer survivors with a HICU change (decrease or increase) were less educated than individuals without any change in HICU (respectively 64.9% and 64.6% had a tertiary level against 71.0% in the reference group). Both groups have also more frequently changed jobs and/or employer after diagnosis, particularly those who have experienced a HICU decrease (39.1% changed jobs against only 19.2% in individuals of the reference group).

In addition, cancer survivors who had a HICU decrease were most of the time of a female gender (87.5% were female *versus* 77.1% in the reference group) and worked more frequently with fixed term contracts at diagnosis (12.8% *versus* 6.9%). As expected, working time reduction was correlated with a decrease in HICU. However, this association was not exclusive: more than half (53%) of survivors with a HICU decrease, did not benefit from any reduction in working time after the diagnosis (and a quarter of cancer survivors having had a HICU increase have benefited from a working time reduction after diagnosis). Finally, survivors with a HICU increase were less likely to experience an adverse cancer event after diagnosis (only 14.9% *versus* 20.9% among those without any HICU change).

### Factors associated with a decrease in HICU

As the Heckman model presented in Table 3 indicates, the use of a selection equation for missing data to correct estimators in the second equation (which served to calculate the decrease in HICU) was relevant since the LR test about the independence of equations (rho = 0) was significant. Once the variables introduced in the selection equation were accounted for, most factors remained significant to explain the decrease in HICU. Thus, being a woman, being single, having dependent children, being a renter, and living in a rural area were the socio-demographic characteristics positively associated with a decrease in HICU. Survivors with high HICU at diagnosis were also more likely to report lower income at survey. In addition, having a part-time job at diagnosis, being a craftsman, a shopkeeper or a business owner, changing job and/or employer, and reducing one’s working time were associated with a decrease in HICU. Finally, undergoing chemotherapy after diagnosis was significantly associated with a decrease in HICU.

**Table 3:** Factors associated with a decrease in HICU.

		Model 1. Simple probit with no selection equation (N = 1,483^#^)		Model 2. Probit with Heckman model to account for potntial selection bias (N = 1,636)	
Log-likelihood		-596.450		∅	
Athrho (p value)		∅		-1.778 (0.001)	
		**Coefficient**	**P value**	**Coefficient**	**P value**
**Gender and tumor**					
	Male & any tumor site	-0.374	0.004	-0.339	0.002
	Female & female-specific tumor site	-0.128	0.210	-0.145	0.074
	Female & non-gender-specific tumor site	*REF*		*REF*	
**Age at diagnosis**					
	< 40	-0.175	0.201	-0.311	0.011
	> = 40 and < 50	-0.083	0.429	-0.164	0.071
	> = 50	*REF*		*REF*	
**Marital status at survey**					
	In a relationship	-0.594	< 0.001	-0.524	< 0.001
	Single	*REF*		*REF*	
**Dependent children at survey**					
	Yes	0.309	0.002	0.309	0.001
	No	*REF*		*REF*	
**Decrease in CU between diagnosis and survey**					
	Yes	-0.198	0.060	-0.174	0.038
	No	*REF*		*REF*	
**Educational level at diagnosis**					
	Secondary or less	0.176	0.093	0.263	0.004
	Tertiary	*REF*		*REF*	
**Housing tenure status**					
	Homeowner	*REF*		*REF*	
	Renter	0.321	0.003	0.219	0.019
**Place of residence**					
	Rural area	0.228	0.017	0.166	0.033
	City	*REF*		*REF*	
**Sector of activity at diagnosis**					
	Public	0.077	0.455	-0.063	0.506
	Private	*REF*		*REF*	
**Socio-professional category at diagnosis**					
** **	Independent farmer, business owner	0.206	0.445	0.302	0.132
	Craftsman, shopkeeper	0.591	< 0.001	0.479	0.001
	Manager (white collar)	*REF*		*REF*	
	Intermediate occupation	-0.088	0.492	-0.160	0.113
	Employee	0.115	0.402	-0.049	0.678
	Blue collar worker, farm worker	0.256	0.121	0.104	0.445
**Working time at diagnosis**					
	Part-time	0.269	0.012	0.202	0.023
	Full-time	*REF*		*REF*	
**HICU at diagnosis**					
	High (> 3^rd^ quartile)	*REF*		*REF*	
	Intermediate (q1-q3)	-0.523	< 0.001	-0.430	< 0.001
	Low (< 1^st^ quartile)	-1.559	< 0.001	-1.261	< 0.001
**Change of job and/or employer after diagnosis**					
	Yes	0.244	0.012	0.193	0.015
	No	*REF*		*REF*	
**Working time reduction after diagnosis**					
	Yes	0.465	< 0.001	0.337	< 0.001
	No	*REF*		*REF*	
**Duration of 1st sick leave (in months)**					
		0.007	0.060	0.007	0.034
**Chemotherapy at diagnosis**					
	Yes	0.215	0.021	0.160	0.038
	No	*REF*		*REF*	
**Adverse cancer event between diagnosis and survey**					
	Yes	-0.187	0.091	-0.069	0.474
	No	*REF*		*REF*	

^#^ Non-responses were excluded from model 1.

Note to the reader: All other factors being equal, and regardless of whether the tumor site was gender-specific or not, men were significantly less likely to experience a decrease in HICU than women.

After we estimated potential bias due to non-response, other factors became significantly associated with a decrease in HICU: being under 40 years of age was negatively associated with a decrease in HICU, whereas low educational level was positively associated with it.

### Perceived variation in household income

**Fig 2:**
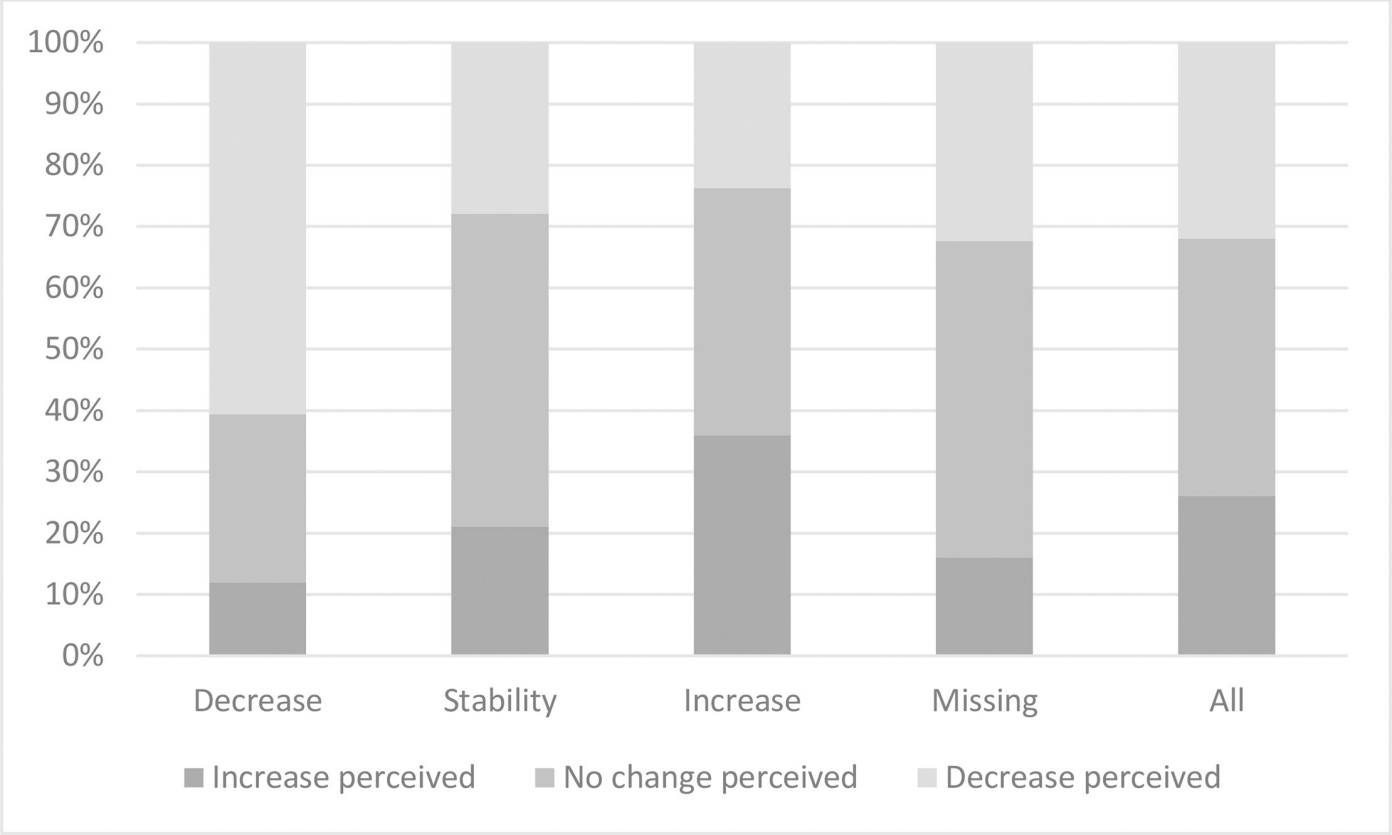
Perceived variation in household income according to actual variation (in %) (N = 1,636).

Five years after diagnosis, 31.6% of participants reported a perceived decrease in household income, whereas 25.8% reported a perceived increase and 41.4% reported no perceived change. Six in ten survivors who experienced an actual decrease in HICU reported a perceived decrease in household income, whereas around one in ten reported a perceived increase (Fig 2). The largest difference between the two indicators was found in survivors who experienced an actual increase in HICU: only 35.8% of them reported a perceived increase in household income.

In addition, almost two in three survivors (63.8%) who experienced an actual decrease in HICU reported a non-comfortable economic situation at survey, whereas less than one in two survivors in the other variation groups reported a non-comfortable situation (40.9% of survivors who experienced no change in HICU and 46.3% of survivors who experienced an increase in HICU). Lastly, 46.1% of survivors who experienced an actual decrease in HICU stated that cancer had contributed to reducing their professional income, whereas around a quarter of survivors who did not experience an actual decrease in HICU stated that cancer had done so (26.8% of survivors who experienced no change in HICU and 24.5% of survivors who experienced an increase in HICU).

## Discussion

This study explored income change in a representative sample of cancer survivors five years after diagnosis. Our three main findings are as follows: 1/ more than one in six cancer survivors still working five years after diagnosis (17.6%) reported a decrease in income; 2/ the decrease in HICU was greater in women than in men regardless of tumour site; and 3/ low socio-economic status was significantly associated with a decrease in HICU.

While the French National Health Insurance Fund covers most cancer-related costs, more than one in six cancer survivors in our study reported an income at survey that was at least ten percent lower than that reported at diagnosis. Our initial assumption was that any decrease in HICU experienced by those survivors was the result of working time reduction. However, although an association was found between working time reduction and decrease in HICU, no endogenous link could be detected between these two variables. We concluded from this that working time reduction could not fully explain lower HICU in survivors who were still working five years after diagnosis.

### Strengths & limitations of the study

To our knowledge, this is the first study to investigate income change in a representative sample of cancer survivors in France. By drawing on the VICAN5 survey, we were able to include in our analyses a wide range of medical, socio-economic, and professional data that were collected over a period of five years. Our study is also one of the first to analyse the medical and socio-demographic characteristics of cancer survivors in conjunction with perceptions of income change over time.

Nevertheless, our study also has limitations. The possibility that the French population unaffected by cancer also experienced economic decline during the period under study cannot be excluded. In the absence of a general population cohort followed for up to 5 years for the study of income change, no such comparison could be made. Moreover, no survey conducted within a specific group like people with other severe diseases had a comparable design, over a five-year period. However, this study constitutes a first step in the description of incomes after a chronic disease in scientific literature and must be followed by other future studies.

Another limitation was that a lot of data were missing due to non-response to the income question. Self-employed individuals were especially likely not to answer this question, which may be explained by the fact that their income varies according to their monthly activity. Nevertheless, our results are consistent with the literature: non-response to questions on income is quite common in French medical studies [30], ranging from 4.6% to 23.9% depending on the topic covered. In view of this, investigators generally include questions on income that are answerable with a range of values, like what we did in our study. They also often employ the same method we did to account for missing data due to non-response [23]. This well-proven method was relevant in our study, as shown in Table 3.

### Women were more likely than men to experience a decrease in HICU regardless of tumour site

In France, the average wage differential between men and women is estimated at around 20% [31,32]. Moreover, men tend to be the main breadwinner in the household, whereas women tend to be the main caregiver. In view of this, we supposed that in couples, the female partner of a severely ill male survivor would choose to work less in order to care for her partner and that, conversely, the male partner of a severely ill female survivor would work more in order to offset his partner’s loss of income. Our hypothesis, then, was that in cancer-affected households, the decrease in HICU would be more important when the male partner was diagnosed with cancer than when the female partner was affected by the disease. This hypothesis was supported by other studies. For instance, in a study conducted in Norway two and five years after diagnosis [33], women’s earnings decreased regardless of whether they or their partner was diagnosed with cancer, whereas men’s earnings decreased only if they were affected by the disease.

Surprisingly, our results regarding gender effect contradicted our hypothesis (as did our descriptive analyses). A consistent effect of gender on the decrease in HICU was indeed found in cancer-affected households, but the decrease in HICU was always more important when the female partner was affected by cancer, and this regardless of tumour site. Sensitive analysis confirmed these results: when breast cancer survivors were excluded from the analysis, the gender effect remained significant. In addition, it has been shown that female survivors took significantly longer sick leaves and reduced their working hours to a greater extent than male survivors [34], which increased the gap between female and male survivors. These findings raised an important question regarding the division of labour in the household: given that men earn more than women on average, do cancer-affected households adopt a rebalancing strategy that causes women to invest less in their career compared to men?

#### Being in a relationship facilitates economic adjustment in female survivors

When the regression was stratified by gender, the effect of marital status was significant only for women. Indeed, while single female survivors were more likely to experience a decrease in income compared to female survivors in a relationship, this association was not found in male survivors.

### Some socio-professional categories were more likely than others to experience a decrease in HICU

Craftsmen and shopkeepers experienced a greater decrease in HICU than other socio-professional categories. This finding may be explained by the fact that unlike salaried survivors, self-employed survivors are not entitled to sick-leave benefits, even though their cancer-related costs (from treatment to transportation costs) are covered by the French National Health Insurance Fund. Similar results have been found in studies conducted elsewhere. Thus, in Canada, a study has shown that self-employed survivors experience a greater decrease in income than salaried ones six months after cancer diagnosis [5]. Similarly, a Norwegian study has found that the negative impact of cancer is greater on self-employed survivors one year after diagnosis [35]. Our study adds to this literature by showing that self-employed survivors continue to suffer economic decline five years after diagnosis.

### Social vulnerability was associated with a greater decrease in HICU

The only information available on physical status was comorbidity index at diagnosis, which was not found to be associated with decrease in HICU. By contrast, we had access to a lot of information on the social context and socio-economic characteristics of cancer survivors.

Based on these data, we found that the population most concerned by a decrease in HICU was also the most vulnerable on the labour market. They were characterized by: low educational level, age over 50, being single, having dependent children, living in a rural area, being a renter, and having a part-time job at diagnosis. These findings are consistent with the literature, with several authors reporting that low educational level is associated with a decrease in income [2,6,15,16]. Note that individuals who changed job and/or employer were likely to experience a decrease in HICU, suggesting that change of job generally entailed working time reduction, and therefore resulted from job loss rather than internal promotion. Other authors have observed that cancer can fuel feelings of vulnerability [36], including in the workplace [37], which points to the existence of a double-penalty effect.

#### The impact of cancer on survivors with high HICU at diagnosis differed according to educational level

Cancer survivors whose income at diagnosis was higher than that of three-quarters of the study population were more likely to experience a decrease in HICU. The likely reason is that their professional income was more variable (i.e., more likely to derive in part from a bonus system) and less likely to be offset by social benefits. Among these specific survivors, educational level was strongly associated with a decrease in HICU: 42.7% of those with less than secondary education experienced a decrease in HICU compared to 26.1% of those with tertiary education. The difference in decrease between high educated and low educated survivors was even greater among salaried workers than among self-employed ones. In addition, a greater number of low educated survivors reported a reduction in working time after diagnosis. There are two possible explanations for these results: the coping strategies of cancer survivors vary according to educational level, and educated survivors have more difficulty in finding a new job than high educated survivors.

### Subjective measures of income variation and economic well-being were a useful complement to objective measures

Income variation subjectively measured at survey differed significantly from income variation objectively measured between diagnosis and survey. Indeed, 31.6% of cancer survivors still working five years after diagnosis reported a perceived decrease in household income, when in fact only 17.6% of those survivors experienced an actual decrease in HICU. This over-representation of income reduction in subjective data may reflect a substantial decrease in purchasing power. Thus, an increase in cancer-related expenses such as out-of-pocket fees [38] may have prompted cancer survivors with stable HICU to report a perceived decrease in household income. Moreover, nearly half of cancer survivors stated that cancer had contributed to reducing their professional income. There are many possible explanations for this gap between the perceived and actual impact of cancer on economic well-being: it may be, for instance, that the partner of a cancer survivor was *compelled* to compensate for the latter’s loss of income. Given the high proportion of cancer survivors who feel discriminated or penalized at work because of the disease [21,34,39], survivors’ perception that cancer had a negative impact on their income may have been induced by the feeling of having lost a promotion opportunity. Only the confrontation between perception of decrease and actual measured decrease of HICU allows us to assume a potential response shift bias.

Perception of economic well-being is another useful indicator for capturing the impact of cancer, as it allows investigators to account for survivors who choose to reduce their working hours, and hence their professional income, in order to have better living conditions. In our study, however, a decrease in HICU was found to be strongly associated with a perception of non-comfortable economic situation, suggesting that a decrease in HICU had a negative impact on the living conditions of cancer survivors.

## Conclusion

Our study of income change among cancer survivors five years after diagnosis has shown that inequalities in economic well-being persist long after diagnosis in France, and this despite the fact that most cancer-related costs are covered by the French National Health Insurance Fund. By analysing the medical, socio-demographic, and socio-economic characteristics of cancer patients in conjunction with perceptions of income change, we were able to trace the differentiated evolution of cancer survivors’ income over time, but also to catch a glimpse into some of the strategies put in place by the latter to cope with the disease.

Further research is needed to fully assess the impact of cancer on the economic lives of survivors. For instance, a study comparing a group of survivors with a control group from the general population could help shed light on the association between the disease and income reduction long after diagnosis. Moreover, a comparative study of survivors in France with survivors in countries with different insurance coverage schemes could contribute to designing public health policy that better addresses the economic problems caused by the disease. Gender-specific studies should also be conducted to help develop a more refined understanding of the differential impact of cancer on women and men. Lastly, more attention should be paid to cancer patients with low socio-economic status in order to help reduce inequalities in post-diagnosis living conditions.

## Supporting information

S1 File Dataset. SAS 9.4 Table.
(SAS7BDAT)

## Abbreviations

HICU: Household income per consumption unit
